# Workplace Injury and Mental Health Outcomes

**DOI:** 10.1001/jamanetworkopen.2024.59678

**Published:** 2025-02-13

**Authors:** Anthony Wightman, Justin P. Gawaziuk, Rae Spiwak, Lauren Burton, Brenda Comaskey, Dan Chateau, Jordan Nantais, Thomas Turgeon, Jitender Sareen, James Bolton, Allen Kraut, Sarvesh Logsetty

**Affiliations:** 1 Department of Surgery, Max Rady College of Medicine, University of Manitoba, Winnipeg, Manitoba, Canada; 2 Australian Institute of Health and Welfare, Canberra, Australia; 3 Department of Psychiatry, Max Rady College of Medicine, University of Manitoba, Winnipeg, Manitoba, Canada; 4 Department of Community Health Sciences, Max Rady College of Medicine, University of Manitoba, Winnipeg, Manitoba, Canada; 5 Department of Psychology, University of Manitoba, Winnipeg, Manitoba, Canada; 6 Department of Internal Medicine, Max Rady College of Medicine, University of Manitoba, Winnipeg, Manitoba, Canada

## Abstract

**Importance:**

Workplace injury is a widespread problem that impacts mental health and quality of life and places a substantial burden on employers and the health care system.

**Objective:**

To determine whether mental disorder rates differ following workplace injury compared with injuries outside the workplace.

**Design, Setting, and Participants:**

This retrospective cohort study assessed individuals hospitalized for an injury requiring surgery between January 1, 2002, and December 31, 2018, with a 2-year follow-up period using population-based administrative data in Manitoba, Canada. Analyses were completed June to July 2021. This study compared 2 cohorts: individuals with a workplace injury matched 1:5 on sex, age, geographical region, and surgical procedure code with individuals with a nonworkplace injury in the general population.

**Exposure:**

Traumatic physical injury that required surgery with anesthetic.

**Main Outcomes and Measures:**

The outcome of interest was a diagnosis of mental disorder (anxiety, depression, substance abuse, suicide attempt, and any mental disorder), measured 2 years prior to and following injury.

**Results:**

In this cohort study, 7556 individuals (mean [SD] age, 44.8 [13.3] years; 5721 [75.7%] male; 4624 individuals [61.2%] with urban residence; 4545 individuals [60.1%] with low income) with a workplace injury were compared with 28 901 matches from the general population. The workplace cohort had lower rates of all mental disorders (anxiety: adjusted rate ratio [ARR], 0.82; 95% CI, 0.77-0.87; depression: ARR, 0.78; 95% CI, 0.72-0.84; substance abuse: ARR, 0.63; 95% CI, 0.55-0.72; suicide attempt: ARR, 0.28; 95% CI, 0.11-0.70; and any mental disorder: ARR, 0.82; 95% CI, 0.78-0.86; all *P* < .0006) before their injury and for depression (ARR, 0.89; 95% CI, 0.82-0.95) and substance abuse (ARR, 0.83; 95% CI, 0.73-0.94) after their injury. The group × care period interaction term was significant for anxiety (*P* < .0001) and any mental disorder (*P* < .0001), suggesting that individuals with workplace injuries had worse mental disorder outcomes over time than individuals with nonworkplace injuries.

**Conclusions and Relevance:**

This cohort study found that the mental health trajectory from the preinjury to postinjury period was worse for individuals with a workplace injury compared with those injured outside the workplace. These findings suggest that there may be features unique to the workplace and/or injury claims and compensation processes that contribute to this pattern, which warrant further examination.

## Key Points

**Question:**

How do mental health outcomes for individuals with a workplace injury compare with mental health outcomes for individuals with a nonworkplace injury?

**Findings:**

In this cohort study of 7556 individuals with workplace injury matched with 28 901 individuals with nonworkplace injury, rates of anxiety and any mental disorder worsened from the preinjury to postinjury period for individuals with a traumatic physical workplace injury compared with individuals with a nonworkplace traumatic physical injury.

**Meaning:**

These findings suggest that there may be factors unique to the workplace and/or injury claims and compensation structures that impact mental health following a workplace injury and these should be considered in interventions and postinjury care.

## Teaser

This cohort study examines mental disorder rates 2 years before and after injury among individuals with traumatic physical workplace injury compared with outside individuals injured outside the workplace.

## Introduction

Physical injury in the workplace is a widespread and significant problem; it is estimated that one-fifth of global deaths and nearly one-third of disability adjusted life-years are attributable to workplace injuries.^1^ Injury causes both immediate and long-term physical consequences, including chronic pain and illness, discomfort, reduced mobility, and limitations to self-care.^2,3^ Equally devastating and pervasive are the mental health consequences of workplace injury (WPI). WPI and nonworkplace injury are associated with mental disorders, including anxiety, depression, substance use disorders, and suicidality,^2,3,4,5^ which are mediated by persistent physical symptoms, time unable to work, gender, and socioeconomic status.^6,7^ Depression in injured workers is associated with delayed return to work^8^ and has long-term impacts.^9^ These effects are exacerbated in WPI due to lost income, the requirement to return to the workplace where the injury occurred, and difficulty navigating compensation bureaucracies.^10^ In 2022, there were 348 747 lost-time injury claims in Canada,^11^ amounting to approximately 1.8% of employed workers.^12^ Mental disorders due to WPI are a persistent and costly issue: they affect injured workers’ quality of life and impose costs on injured workers, their employers, and the health care system.

Few studies have examined mental health outcomes following WPI, and there are significant gaps in the literature. One large sample study found 45% greater odds of depression in injured workers compared with noninjured workers.^13^ Other research^14,15^ found greater risk of psychiatric disorders for individuals with WPI compared with those with injuries outside the workplace. Where studies have examined mental health outcomes following WPI, they are limited by small sample size,^16^ lack of comparison group,^5,17^ comparison to a noninjured population,^13^ only examine traumatic brain injury,^18^ or do not include preinjury mental health.^14,15^ To address these gaps, we conducted a longitudinal retrospective cohort study using linked population-based administrative data to examine whether WPI are associated with worse mental disorder rates than injuries in the general population (GPI) while accounting for preinjury mental health. Given the challenges faced by injured workers, our hypothesis is that mental health outcomes will be worse for those with WPI compared with GPI. If our hypothesis is correct, findings from this study can inform postinjury mental health care, injury prevention programs, and assessment for workers’ compensation for mental health conditions.

## Methods

This cohort study was approved by the Health Research Ethics Board at the University of Manitoba and the Provincial Health Research Privacy Committee. The requirement for informed consent was waived due to the use of aggregate, anonymized data. Reporting for this study follows the Reporting of Studies Conducted Using Observational Routinely-Collected Health Data (RECORD) guideline.

### Overview

This retrospective matched cohort study examined postinjury mental disorders in WPI compared with GPI. Eligible Manitoba, Canada, Worker’s Compensation Board (WCB) injury claims were linked to health and social administrative data housed at the Manitoba Centre for Health Policy (MCHP) to examine the association of WPI (compared with GPI) with mental health outcomes in the context of preexisting mental health.

### Data Sources

The WCB of Manitoba covers approximately 77% of the provincial workforce^19^ and, as the sole provider of workers’ compensation in Manitoba, reimburses medical costs for accepted medical claims. WCB data includes type of injury and medical and hospital diagnoses and procedures related to the injury. The MCHP Population Health Research Data Repository contains numerous administrative datasets with anonymized information on individuals in Manitoba. The WCB database was linked to the following repository datasets using a unique scrambled identification number^20^: Medical Services (physician billing), Hospital Abstracts, Manitoba Health Insurance Registry, Vital Statistics Mortality, and Canada Census.^20,21^

### Study Population

#### WPI Cohort

Individuals aged at least 18 years with a WCB time claim for an injury that required hospitalization (ie, a WPI) between January 1, 2002, and December 31, 2018 (with the date of admission serving as the index date), were identified within the WCB database and included if they met the following criteria: first-time WPI that required a general anesthetic surgical procedure, valid personal health information number (PHIN), Manitoba address 1 year before the index date, and at least 30 days of Manitoba health insurance coverage after the index date. The last date of inclusion for data was November 30, 2020. The criteria for surgical procedures to require general anesthetic reduced variability, as only individuals who were seriously injured at a discrete point in time and required an invasive therapeutic procedure with general anesthetic were included. This reduced the overall number of eligible individuals; however, the included injuries likely had a significant physiologic impact on the injured individuals. Exclusion criteria were: military personnel (due to lack of Manitoba PHIN and the so-called healthy warrior effect^22^ confounding mental disorders and the healing process^23^), individuals with traumatic brain injuries^24^ and burns^25^ (due to confounding of mental disorder development based on the mechanism of injury and course of healing), and individuals with repetitive strain injuries and chronic conditions (as these injuries do not occur at a discrete point in time).

#### GPI Cohort

The comparison GPI cohort included individuals aged at least 18 years who had a first-time injury requiring surgery with anesthetic that was not work-related, a valid PHIN, a Manitoba address for 1 year prior to the index date, and minimum 30 days of Manitoba health insurance after the index date. The WPI cohort was matched up to 1:5 with the GPI cohort on the following: age at index date (±5 years), sex (male, female), geographic region (residential postal code at index date), and, to control for injury severity, the surgical procedure tariff code (eMethods in Supplement 1).

### Outcome Variables

Mental disorder diagnoses were examined 2 years before and after the index date. The presence of mental disorders was assessed using *International Classification of Diseases, Ninth Revision, Canadian Modification *(*ICD-9-CM*) and *International Statistical Classification of Diseases and Related Health Problems, Tenth Revision, Canada *(*ICD-10-CA*) codes: anxiety (*ICD-9-CM*: 300.0, 300.2, 300.3; *ICD-10-CA*: F40, F41.0, F41.1, F41.3, F41.8, F41.9, F42, F431); depression (*ICD-9-CM*: 296.2–296.3, 296.5, 300.4, 309, 311; *ICD-10-CA*: F31.3-F31.5, F32, F33, F341, F380, F381, F432, F438, F530); and substance use disorders (*ICD-9-CM*: 291, 292, 304, 305, 303; *ICD-10-CA*: F10-F19, F55). Hospitalized suicide attempts were defined as any E950 *ICD-9-CM* code. *Any mental disorder* combined anxiety, depression, substance use disorders, and hospitalized suicide attempt into 1 variable. One or more outpatient visits (physician billing) and/or 1 or more hospitalizations during the preindex or postindex periods were considered a mental disorder diagnosis. Coding for mental disorders of interest was based on diagnostic definitions for the Manitoba population, as published previously by the study team.^26,27^

### Statistical Analysis

Data analysis followed a similar framework previously used by our team^28^ and the WCB analysis strategy. Comparative statistical analysis was conducted to identify differences between the cohorts and descriptive statistics were calculated using *t* tests (continuous variables) and χ^2^ tests (categorical variables). Contingency tables were generated to examine mental disorder diagnoses before and after the index date for WPI compared with GPI, resulting in unadjusted rate ratios (URRs). A Bonferroni correction was applied to minimize the risk of type II error from the multiple analyses, and cohorts were considered statistically different from each other when *P* < .0006 (2-tailed). Inverse probability of treatment weighting was used to mitigate the effect of potential bias between study cohorts and calculated by generating propensity scores from a logistic regression model. This model included binary indicators for sex (male, female), geographic region (urban, rural), and income (high, low). Comparisons of individual propensity score distributions showed sufficient overlap. This suggested that inverse probability of treatment weighting would result in a comparable distribution of variables between WPI and GPI and confirmed the feasibility of comparing the cohorts.^29,30^

A generalized estimating equation (GEE) was used to account for within-person correlation of the data and for repeat entries from the same individual over different points of time.^31^ The GEE calculated adjusted rate ratios (ARRs) for preindex and postindex mental disorders in the WPI group. The GEE included group (cohort), pre–index date period and post–index date period (ie, care period), income, sex, geographic region, and a group × care period interaction term to examine mental disorder rates between the cohorts over time. Average treatment effect (ATE) was estimated and used to weight the GEE. The ATE established an optimal balance between the WPI and GPI cohorts.^30^ A time offset variable (log person-years) was included to account for the effect of time on each participant in the GEE model. Analyses were conducted from June to July 2021 and performed using SAS software version 9.4 (SAS Institute).

## Results

In this cohort study, 7556 individuals (mean [SD] age, 44.8 [13.3] years; 5721 [75.7%] male; 4624 individuals [61.2%] with urban residence; 4545 individuals [60.1%] with low income) with a first-time traumatic physical WPI requiring surgery were matched on sex, age, geographical region, and surgical procedure code to 28 901 individuals with a traumatic physical nonworkplace injury requiring surgery (Figure and Table 1). Table 2 includes summary statistics for WPI cohort injuries and claims. The most frequent causes of injuries were objects in the environment (35.0%), followed by the environment (27.2%) and body position (22.1%). Half (50.4%) of the injuries were on the upper extremity. The most common types of injuries were strains, sprains, and tears (40.3%) followed by fractures and dislocations (25.2%). More than half of the claims were from the service (20.5%), construction (19.9%), and manufacturing (17.6%) industries. Table 3 includes URR of mental disorders for both cohorts in the preinjury and postinjury periods (values for suicide attempt were suppressed due to small cell sizes). In the preinjury period, URRs of all mental disorders were lower in the WPI cohort compared with GPI (anxiety: URR, 0.81; 95% CI, 0.75-0.87; depression: URR, 0.76; 95% CI, 0.70-0.83; substance use: URR, 0.65; 95% CI, 0.57-0.74; any mental disorder: URR, 0.79; 95% CI, 0.75-0.84; all *P* < .0006). After injury, the WPI cohort had lower URRs for depression (URR, 0.89; 95% CI, 0.82-0.96) and substance use disorder (0.87; 95% CI, 0.76-0.99) (both *P* < .0006). This pattern held for the ARRs, which were lower in the WPI cohort for all mental disorders (anxiety: ARR, 0.82; 95% CI, 0.77-0.87; depression: ARR, 0.78; 95% CI, 0.72-0.84; substance abuse: ARR, 0.63; 95% CI, 0.55-0.72; suicide attempt: ARR, 0.28; 95% CI, 0.11-0.70; any mental disorder: ARR, 0.82; 95% CI, 0.78-0.86) before injury and for depression (ARR, 0.89; 95% CI, 0.82-0.95) and substance abuse (ARR, 0.83; 95% CI, 0.73-0.94) after injury (all *P* < .0006) (Table 4). The group × care period interaction term was significant for anxiety (*P* < .0001) and any mental disorder (*P* < .0001), suggesting that WPI had a greater negative association with mental disorders over time than nonworkplace injuries.

**Figure.**
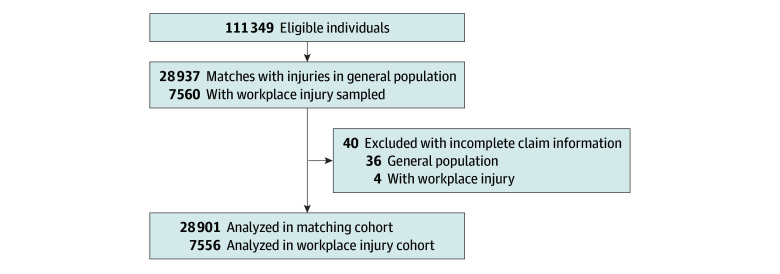
Study Enrollment Flowchart

**Table 1.** Descriptive Characteristics of WPI and GPI Cohorts

Characteristic	Individuals, No. (%)	
	WPI (n = 7556)	GPI (n = 28 901)
Age at index date, mean (SD), y	44.8 (13.3)	45.5 (13.6)
Sex		
Male	5721 (75.7)	21 340 (73.8)
Female	1835 (24.3)	7561 (26.2)
Residence^a^		
Urban	4624 (61.2)	17 090 (59.1)
Rural	2932 (38.8)	11 811 (40.9)
Income^b^		
High	3011 (39.9)	11 007 (38.1)
Low	4545 (60.1)	17 894 (61.9)

Abbreviations: GPI, general population injury; WPI, workplace injury.

^a^ Urban indicates population of 50 000 or more; rural, population fewer than 50 000.

^b^ Income quintiles were derived from Canada census neighborhood income averages. High income indicates the upper 3 quintiles; low income, lower 2 quintiles.

**Table 2.** Injury Characteristics of Workplace Injury Cohort

Characteristic	Individuals, No. (%) (n = 7556)
Injury cause	
Objects in the environment^a^	2641 (35.0)
Environment (built or otherwise)^a^	2054 (27.2)
Bodily motion or position	1670 (22.1)
Vehicle, mobile equipment, or conveyance	560 (7.4)
Unknown or missing^b^	300 (4.0)
People in environment	272 (3.6)
Animals	59 (0.8)
Injury type	
Strains, sprains, tears	3044 (40.3)
Fractures, dislocations	1904 (25.2)
Cutaneous injury	969 (12.8)
Systemic diseases and disorders (eg, hernia)	877 (11.6)
Other	458 (6.1)
Unknown	304 (4.0)
Anatomical location	
Upper extremity	3807 (50.4)
Lower extremity	1979 (26.2)
Multiple extremities	1063 (14.1)
Trunk	625 (8.3)
Other	49 (0.7)
Head	33 (0.4)
Employment sector	
Service	1550 (20.5)
Construction	1501 (19.9)
Manufacturing	1330 (17.6)
Trade	979 (13.0)
Self-insured	919 (12.2)
Transportation, communications, and storage	769 (10.2)
Agriculture and forestry	187 (2.5)
Mines, quarries, and oil wells	135 (1.8)
Public administration	116 (1.5)
Optional coverage	70 (0.9)

^a^ Categories collapsed due to small cell sizes.

^b^ Missing complete claims information in 86 individuals (1.1% of WPI cohort).

**Table 3.** URR of Mental Disorders in WPI vs GPI Cohorts 2 Years Before and After Injury

Mental disorder	2 y preinjury			2 y postinjury		
	No. (%)		URR (95% CI)^a^	No. (%)		URR (95% CI)^a^
	WPI (n = 7556)	GPI (n = 28 901)		WPI (n = 7556)	GPI (n = 28 901)	
Anxiety	1126 (14.9)	5142 (17.8)	0.81 (0.75-0.87)^b^	1284 (17.0)	4765 (16.5)	1.04 (0.97-1.11)
Depression	761 (10.1)	3694 (12.8)	0.76 (0.70-0.83)^b^	824 (10.9)	3506 (12.1)	0.89 (0.82-0.96)^b^
Substance use	254 (3.4)	1468 (5.1)	0.65 (0.57-0.74)^b^	288 (3.8)	1262 (4.4)	0.87 (0.76-0.99)^b^
Suicide attempt	NA^c^	NA^c^	NA^c^	NA^c^	NA^c^	NA^c^
Any mental disorder	1729 (22.9)	7872 (27.2)	0.79 (0.75-0.84)^b^	1907 (25.2)	7371 (25.5)	0.99 (0.93-1.06)

Abbreviations: GPI, general population injury; NA, not available; URR, unadjusted rate ratio; WPI, workplace injury.

^a^ Adjusted for sex, geographic region, and income.

^b^ *P* < .0006.

^c^ Cells with fewer than 5 individuals were suppressed.

**Table 4.** ARR of Mental Disorders in WPI vs GPI Cohorts Over Time

Mental disorder	2 y preinjury, ARR (95% CI)^a^	Group × care period, *P* value	2 y postinjury, ARR (95% CI)^a^
Anxiety	0.82 (0.77-0.87)^b^	<.0006	1.00 (0.95-1.06)
Depression	0.78 (0.72-0.84)^b^	.0009	0.89 (0.82-0.95)^b^
Substance use	0.63 (0.55-0.72)^b^	.001	0.83 (0.73-0.94)^b^
Suicide attempt	0.28 (0.11-0.70)^b^	.71	0.36 (0.13-1.02)
Any mental disorder	0.82 (0.78-0.86)^b^	<.0006	0.96 (0.92-1.01)

Abbreviations: ARR, adjusted rate ratio; GPI, general population injury; WPI, workplace injury.

^a^ Adjusted for sex, geographic region, and income.

^b^ *P* < .0006.

## Discussion

This retrospective cohort study found, after adjusting for preinjury mental disorders, that traumatic physical WPI were associated with a worse mental health trajectory from before injury to after injury compared with traumatic physical nonworkplace injuries. This finding supports the hypothesis that a WPI has a greater negative association with mental disorder rates than nonworkplace injuries. This study advances the literature by comparing large population-based cohorts of injured populations, both in and outside the workplace, and their mental health status from the 2 years prior to injury up to 2 years after injury. In this study, the WPI group had lower rates of mental disorders than the GPI group in the preinjury period. This may be explained by the fact that employed individuals tend to have lower rates of mental disorders compared with the general population.^32,33^ While lower rates of mental disorders persisted for WPI following injury, the interaction term for group × care period was significant for anxiety and any mental disorder, indicating that mental health for the WPI group declined over time from the preinjury to postinjury period, relative to the GPI group. The finding that mental health declined from preinjury to postinjury for those with a WPI has been found in other research. A study by Dersh et al^33^ compared rates of psychiatric disorders in a WPI cohort with general population estimates and found lower rates before injury and elevated rates after injury in the WPI group. However, the study used a structured clinical interview and measured preinjury and postinjury mental health at discrete points in time. A study by O’Hagan et al^5^ found that there were higher rates of mental health problems with onset in the postinjury period compared with preinjury onset, although findings were obtained through a cross-sectional self-report telephone survey. Other research found no difference in self-reported psychological distress among workers with WPI compared with workers with a nonworkplace injury^34^; however, mental health may be more affected for those with more severe injuries.^14^ Factors associated with increased risk of suicide among injured workers include injury severity and length of hospitalization.^35^ While our study did not find any significant difference in rate of suicide attempt after injury between the cohorts or from the preinjury to postinjury period for WPI, other large sample studies have found an elevated risk of suicide after injury.^36,37^ In addition, our study found an elevated risk of substance use disorder following workplace injury, as has been found in another large sample study.^36^

The implications for these findings are substantial. First, research has shown that injured workers incur higher costs for mental health treatment than noninjured workers.^13^ For low-income injured workers, there may be an increased risk of mental disorders^34^ after injury due to pressure to return to work prematurely^38^ and/or the need to return to the workplace where the injury occurred.^39^ Conversely, individuals injured in the workplace may experience further burden on mental health due to delayed return to work^39^ and bureaucratic stressors of the compensation system.^10^ Second, those working in low-skill (eg, service) or physically demanding (eg, construction, manufacturing, agriculture) occupations may face reduced long-term compensation and socioeconomic status due the inability to return to work and/or fewer job prospects. Third, the effects of injury on health and well-being can be long-term. Dong et al^2^ found that self-reported mental and physical health, well-being, and functional limitations were worse for injured workers at a mean of 10 years after their injury. Injured workers are at increased risk for reinjury for up to 4 years,^40^ and those who experienced high levels of stress from the compensation process are at increased risk of depression and anxiety 6 years after injury.^10^ In addition, severe injuries may have a longer-term negative impact on mental health; O’Donnell et al^41^ found an increased risk of depression, substance use, and anxiety up to 6 years after a hospitalized injury and Halvachizadeh et al^42^ reported that more than half of multiple-trauma patients had psychiatric disorders up to 20 years after injury. The impact of these injuries is further compounded by an increased risk of disability following a postinjury psychiatric disorder^41^ and the challenges faced by workers after injury

### Strengths and Limitations

A key strength of this study is the use of whole-population administrative data, which reduces the risk of bias due to participant selection, self-report, and patient recollection and avoids diagnostic criteria inconsistency.^2,15^ Other studies on mental disorders following WPI use surveys or follow-up interviews.^2,4,9,15,39^ Additionally, population-based data allowed for the examination of multiple outcomes; many studies only include a single mental disorder.^13,14^ Another strength of this study was the inclusion of individuals injured outside the workplace, comparing 2 injured cohorts that differed in context of the injury. Other studies with a large sample size used comparison groups from a general uninjured population or noninjured workers, which does not allow for a comparison of WPI to non-WPI mental disorder rates.^13,43^ A follow-up period of 2 years after injury allowed time for postinjury mental disorders to develop but not so much time that disorders unrelated to the injury developed. This study’s follow-up advantage was compounded by the study only assessing individuals with first-time injuries. This allowed for a clearer assessment of the association between WPI and mental disorders, which could be confounded by multiple injuries of various intensity. The inclusion criteria that procedures required general anesthetic reduced variability in injury type and ensured that the severity of the injuries was comparable between the WPI and GPI cohorts. Furthermore, this study used robust statistical analysis; the GEE considered ATE in its calculation of the ARR. Using ATE balanced the associations of injury between the WPI and GPI cohorts, allowing for an unbiased estimate of mental disorder rates.

This study has some limitations. The categories for injury source and location were based on WCB data and do not directly align with the more widely used *ICD-9-CM* and *ICD-10-CA* coding systems for injury. We were unable to determine the employment status of individuals in the GPI cohort. In addition, the GPI cohort may include individuals with a WPI who did not receive coverage from the WCB. Other limitations of this study relate to mental disorder diagnoses. Diagnoses in administrative data reflect treatment prevalence rather than symptoms or illness severity, and physicians may not use the same criteria to make diagnoses. Additionally, this study was unable to adjust for Axis II personality disorders (eg, borderline personality disorder), as these disorders arise over the course of a lifetime and not due to a specific incident. Suicide attempt could not be presented as a relative rate due to low numbers. Furthermore, this study was unable to assess posttraumatic stress disorder as an outcome as the second decimal place to differentiate posttraumatic stress disorder from other anxiety disorders was not available.

## Conclusions

This retrospective cohort study examined mental disorder rates in WPI compared with GPI over time. While rates of mental disorders were lower for WPI before and after injury, this study found that mental disorder rates following a WPI were measurably worse compared with a population with nonworkplace injuries, addressing an important gap in the literature. Future directions for research include further examination of risk factors associated with increased mental disorders in individuals with WPI, investigating why preinjury mental disorder rates are lower in those with a WPI compared with GPI, assessing the dataset for outcomes among specific occupational sectors, studying factors that mediate mental disorders, and tailoring interventions to improve post-WPI mental disorder rates and return to work. Injuries exact a significant physical and mental toll on workers and nonworkers alike leading to individual, financial, and systemic costs. Early detection and treatment of mental disorders following traumatic WPI may reduce the long-term impact and burden of these conditions on individuals, the workplace, and society.

## Notes

Supplement 1. **eMethods.** List of Surgical Procedure Codes

Supplement 2.
Data Sharing Statement

## References

[1] World Health Organization. WHO/ILO Joint Estimates of the Work-related Burden of Disease and Injury. Accessed January 14, 2025. https://www.who.int/teams/environment-climate-change-and-health/monitoring/who-ilo-joint-estimates

[2] Dong XS, Wang X, Largay JA, Sokas R. Long-term health outcomes of work-related injuries among construction workers—findings from the National Longitudinal Survey of Youth. Am J Ind Med. 2015;58(3):308-318. doi:10.1002/ajim.22415 25678458

[3] Chin WS, Guo YL, Liao SC, . Suicidality 6 years after occupational injury. J Clin Psychiatry. 2018;79(5):17m11729. doi:10.4088/JCP.17m11729 30192447

[4] Orchard C, Carnide N, Mustard C, Smith PM. Prevalence of serious mental illness and mental health service use after a workplace injury: a longitudinal study of workers’ compensation claimants in Victoria, Australia. Occup Environ Med. 2020;77(3):185-187. doi:10.1136/oemed-2019-105995 31896616

[5] O’Hagan FT, Ballantyne PJ, Vienneau P. Mental health status of Ontario injured workers with permanent impairments. Can J Public Health. 2012;103(4):e303-e308. doi:10.1007/BF03404240 23618646 PMC6974271

[6] Keogh JP, Nuwayhid I, Gordon JL, Gucer PW. The impact of occupational injury on injured worker and family: outcomes of upper extremity cumulative trauma disorders in Maryland workers. Am J Ind Med. 2000;38(5):498-506. doi:10.1002/1097-0274(200011)38:5<498::AID-AJIM2>3.0.CO;2-I 11025491

[7] Sareen J, Erickson J, Medved MI, . Risk factors for post-injury mental health problems. Depress Anxiety. 2013;30(4):321-327. doi:10.1002/da.22077 23408506

[8] von Schroeder HP, Xue CR, Yak A, Gandhi R. Factors associated with unsuccessful return-to-work following work-related upper extremity injury. Occup Med (Lond). 2020;70(6):434-438. doi:10.1093/occmed/kqaa106 32537651

[9] Chu PC, Chin WS, Guo YL, Shiao JSC. Long-term effects of psychological symptoms after occupational injury on return to work: a 6-year follow-up. Int J Environ Res Public Health. 2019;16(2):235. doi:10.3390/ijerph16020235 30650654 PMC6352156

[10] Grant GM, O’Donnell ML, Spittal MJ, Creamer M, Studdert DM. Relationship between stressfulness of claiming for injury compensation and long-term recovery: a prospective cohort study. JAMA Psychiatry. 2014;71(4):446-453. doi:10.1001/jamapsychiatry.2013.4023 24522841

[11] Association of Workers’ Compensation Boards of Canada. National Work Injury, Disease and Fatality Statistics—2020-2022. Accessed October 29, 2024. https://awcbc.org/data-and-statistics/national-work-injury-disease-statistics-program/nwisp-publication

[12] Statistics Canada. Labour force characteristics by sex and detailed age group, annual. Accessed November 5, 2024. https://www150.statcan.gc.ca/t1/tbl1/en/tv.action?pid=1410032701

[13] Asfaw A, Souza K. Incidence and cost of depression after occupational injury. J Occup Environ Med. 2012;54(9):1086-1091. doi:10.1097/JOM.0b013e3182636e29 22929794 PMC4570484

[14] Kim J. Depression as a psychosocial consequence of occupational injury in the US working population: findings from the medical expenditure panel survey. BMC Public Health. 2013;13(1):303. doi:10.1186/1471-2458-13-303 23560685 PMC3635882

[15] Lin KH, Chu PC, Kuo CY, Hwang YH, Wu SC, Guo YL. Psychiatric disorders after occupational injury among National Health Insurance enrollees in Taiwan. Psychiatry Res. 2014;219(3):645-650. doi:10.1016/j.psychres.2014.06.015 24984578

[16] Mason S, Wardrope J, Turpin G, Rowlands A. Outcomes after injury: a comparison of workplace and nonworkplace injury. J Trauma. 2002;53(1):98-103. doi:10.1097/00005373-200207000-00019 12131397

[17] Carnide N, Franche RL, Hogg-Johnson S, . Course of depressive symptoms following a workplace injury: a 12-month follow-up update. J Occup Rehabil. 2016;26(2):204-215. doi:10.1007/s10926-015-9604-3 26324252

[18] Terry DP, Iverson GL, Panenka W, Colantonio A, Silverberg ND. Workplace and non-workplace mild traumatic brain injuries in an outpatient clinic sample: a case-control study. PLoS One. 2018;13(6):e0198128. doi:10.1371/journal.pone.0198128 29856799 PMC5983513

[19] Workers Compensation Board of Manitoba. Workers compensation coverage. Accessed May 12, 2024. https://www.wcb.mb.ca/workers-compensation-coverage

[20] Roos LL, Nicol JP. A research registry: uses, development, and accuracy. J Clin Epidemiol. 1999;52(1):39-47. doi:10.1016/S0895-4356(98)00126-7 9973072

[21] Manitoba Centre for Health Policy. The Manitoba Population Health Research Data Repository. Accessed March 12, 2024. https://umanitoba.ca/manitoba-centre-for-health-policy/data-repository#data-list-and-data-descriptions

[22] Larson GE, Highfill-McRoy RM, Booth-Kewley S. Psychiatric diagnoses in historic and contemporary military cohorts: combat deployment and the healthy warrior effect. Am J Epidemiol. 2008;167(11):1269-1276. doi:10.1093/aje/kwn084 18436536

[23] Enns MW, Mota N, Afifi TO, . Course and predictors of major depressive disorder in the Canadian Armed Forces Members and Veterans Mental Health Follow-up Survey: Cours et Prédicteurs du Trouble de Dépression Majeure Dans l’Enquête de Suivi Sur la Santé Mentale Auprès Des Membres des Forces Armées Canadiennes et des ex-Militaires. Can J Psychiatry. 2021;66(11):971-981. doi:10.1177/0706743720984677 33406886 PMC8649828

[24] Howlett JR, Nelson LD, Stein MB. Mental health consequences of traumatic brain injury. Biol Psychiatry. 2022;91(5):413-420. doi:10.1016/j.biopsych.2021.09.024 34893317 PMC8849136

[25] Logsetty S, Shamlou A, Gawaziuk JP, . Mental health outcomes of burn: a longitudinal population-based study of adults hospitalized for burns. Burns. 2016;42(4):738-744. doi:10.1016/j.burns.2016.03.006 27049068

[26] Enns J, Gawaziuk JP, Khan S, . Mental and physical health outcomes in parents of children with burn injuries as compared with matched controls. J Burn Care Res. 2016;37(1):e18-e26. doi:10.1097/BCR.0000000000000309 26361326

[27] Stone J, Gawaziuk JP, Khan S, . Outcomes in adult survivors of childhood burn injuries as compared with matched controls. J Burn Care Res. 2016;37(2):e166-e173. doi:10.1097/BCR.0000000000000323 26594866

[28] Padalko A, Gawaziuk J, Chateau D, Sareen J, Logsetty S. Social determinants associated with pediatric burn injury: a population-based, case–control study. J Burn Care Res. 2020;41(4):743-750. doi:10.1093/jbcr/iraa045 32352522 PMC7333671

[29] Lix L, Yogendran MSM, Mann J. Defining and validating chronic diseases: an administrative data approach: an update with *ICD-10-CA*. Accessed April 18, 2023. http://mchp-appserv.cpe.umanitoba.ca/reference/ICD10_Final.pdf

[30] Austin PC, Stuart EA. Moving towards best practice when using inverse probability of treatment weighting (IPTW) using the propensity score to estimate causal treatment effects in observational studies. Stat Med. 2015;34(28):3661-3679. doi:10.1002/sim.6607 26238958 PMC4626409

[31] Hanley JA, Negassa A, Edwardes MD, Forrester JE. Statistical analysis of correlated data using generalized estimating equations: an orientation. Am J Epidemiol. 2003;157(4):364-375. doi:10.1093/aje/kwf215 12578807

[32] Bruggeman H, Héroufosse J, van der Heyden J, Smith P. The relationship between mental health and employment: a systematic review of cohort studies. Eur J Pub Health. Published online October 28, 2024. doi:10.1093/eurpub/ckae144.1106

[33] Dersh J, Mayer T, Theodore BR, Polatin P, Gatchel RJ. Do psychiatric disorders first appear preinjury or postinjury in chronic disabling occupational spinal disorders? Spine (Phila Pa 1976). 2007;32(9):1045-1051. doi:10.1097/01.brs.0000261027.28779.52 17450081

[34] Gu JK, Charles LE, Fekedulegn D, Ma CC, Violanti JM, Andrew ME. Occupational injury and psychological distress among U.S. workers: the National Health Interview Survey, 2004-2016. J Safety Res. 2020;74:207-217. doi:10.1016/j.jsr.2020.06.002 32951785 PMC7753949

[35] Kuo CY, Liao SC, Lin KH, . Predictors for suicidal ideation after occupational injury. Psychiatry Res. 2012;198(3):430-435. doi:10.1016/j.psychres.2012.02.011 22436350

[36] Applebaum KM, Asfaw A, O’Leary PK, Busey A, Tripodis Y, Boden LI. Suicide and drug-related mortality following occupational injury. Am J Ind Med. 2019;62(9):733-741. doi:10.1002/ajim.23021 31298756 PMC7485601

[37] Lee HE, Kim I, Kim MH, Kawachi I. Increased risk of suicide after occupational injury in Korea. Occup Environ Med. 2021;78(1):43-45. doi:10.1136/oemed-2020-106687 32796094 PMC7803905

[38] Nosanov LB, Romanowski KS. Firefighter postinjury return to work: a balance of dedication and obligation. J Burn Care Res. 2020;41(5):935-944. doi:10.1093/jbcr/iraa079 32441739

[39] Kendrick D, Dhiman P, Kellezi B, . Psychological morbidity and return to work after injury: multicentre cohort study. Br J Gen Pract. 2017;67(661):e555-e564. doi:10.3399/bjgp17X691673 28630058 PMC5519127

[40] Sears JM, Schulman BA, Fulton-Kehoe D, Hogg-Johnson S. Estimating time to reinjury among Washington State injured workers by degree of permanent impairment: Using state wage data to adjust for time at risk. Am J Ind Med. 2021;64(1):13-25. doi:10.1002/ajim.23200 33210293 PMC7768893

[41] O’Donnell ML, Alkemade N, Creamer MC, . The long-term psychiatric sequelae of severe injury: a 6-year follow-up study. J Clin Psychiatry. 2016;77(4):e473-e479. doi:10.4088/JCP.14m09721 27137435

[42] Halvachizadeh S, Teuber H, Berk T, . Prevalence, injury-, and non–injury-related factors associated with anxiety and depression in polytrauma patients—a retrospective 20 year follow-up study. PLoS One. 2020;15(5):e0232678. doi:10.1371/journal.pone.0232678 32365087 PMC7197792

[43] Haider AH, Herrera-Escobar JP, Al Rafai SS, . Factors associated with long-term outcomes after injury: results of the Functional Outcomes and Recovery after Trauma Emergencies (FORTE) multicenter cohort study. Ann Surg. 2020;271(6):1165-1173. doi:10.1097/SLA.0000000000003101 30550382

