# Supplementary material for: Workplace Injury and Mental Health Outcomes
Source: JAMA Netw Open. 2025 Feb 13;8(2):e2459678. doi: 10.1001/jamanetworkopen.2024.59678 (PMC11826355; doi:10.1001/jamanetworkopen.2024.59678)
Supplement: Supplement 1. — eMethods. List of Surgical Procedure Codes [file jamanetwopen-e2459678-s001.pdf]

Supplemental Online Content

Wightman A, Gawaziuk JP, Spiwak R, et al. Workplace injury and mental health outcomes. *JAMA Netw Open*. 2025;8(2):e2459678. doi:10.1001/jamanetworkopen.2024.59678

eMethods. List of Surgical Procedure Codes

This supplemental material has been provided by the authors to give readers additional information about their work.

© 2025 Wightman A et al. *JAMA Network Open*.

eMethods. List of surgical procedure codes

1088 - Arthroscopy, knee, microfracture
1089 - Knee, additional meniscectomy or meniscal repair
1090 - Knee, debride femoral condyle
1091 - Knee, debride tibial plateau
1092 - Knee, patellar, retinacular release
1094 - Knee, removal of loose body
0256 - Foreign body subcutaneous tissue, complicated, incision and removal
0259/0260 - Debridement of full thickness chronic skin ulcer i.e., neuropathic or vascular, down to fascia, bone and/or muscle
0261 - Major debridement of necrotizing soft tissue infection
0289/0290/0292/0293 - Repair by adjacent tissue transfer
0303/0304/0305 - Split skin grafts
0309 - Full-thickness grafts, free
0343/0349 - Elevation of free island skin and subcutaneous flap and closure of defect
0344 - Reimplantation, digit, with or without vein graft
0350 - Preparation of microvascular recipient site for free island skin subcutaneous flap
0352 - Dressings, 2nd or 3rd degree, initial or subsequent, with anesthetic, small or medium
0358 - Transplantation of free island skin and subcutaneous flap with microvascular anastomosis(es)
0359 - Non burn dressings, major debridement and dressing, with anesthesia (excluding local anesthesia)
0380/0381/0382 - Split skin grafts, creation of recipient area
0387 - Full thickness grafts
0524 - Cutting, division or transection of bone with or without fixation - calcaneum
0525 - Bones, removal of plates, pins, wires, screws, etc.
0530/0531/0536/0537 - Osteotomy, cutting, division or transection of bone
0557/0558/0561 - Excision of bone, single bone/carpus, proximal row/patella, partial or total
0565/0566/0567/0572 - Excision of bone cyst, chondroma or exostosis, fibula, radius, ulna/femur, humerus, other bones
0576/0577 - Osteomyelitis, major, craterization, guttering or saucerization of bone; diaphysectomy, including closed irrigation
0591 - Bone wiring, Kirschner wire, insertion
0617/0618/0620/0624 - Bone graft, including obtaining and placing of graft
0634 - Bone graft, harvesting - iliac bone
0635/0636 - Decompression, spine, multiple vertebrae
0742 - Fractures, spine and trunk, clavicle, open reduction
0782/0787/0790/0794/0801/0805/0810/0811/0816/0819/0823/0830/0844/0848/0854/0868 - Fractures, upper extremity, open reduction
0874/0883/0897/0904/0910/0912/0916/0928/0935/0937/0941/0942/0946/0963/0970/0982/0989 - Fractures, lower extremity, open reduction
1001/1002/1003/1006/1008/1010/1013/1017 - Joints, arthrotomy or capsulotomy
1025 - Diagnostic arthroscopy shoulder (independent procedure)
1027 - Arthroscopy shoulder with therapeutic intervention
1028 - Synovectomy shoulder - complete
1029 - Subacromial decompression
1030 - Distal clavicle excision
1032 - Stabilization for recurrent sternoclavicular, acromioclavicular instability
1033/1034 - Glenohumeral stabilization
1035 - Superior Labrum Anterior - Posterior (SLAP) repair
1036 - SLAP and anterior or posterior glenohumeral stabilization
1037/1038 - Rotator cuff repair and/or SLAP repair

© 2025 Wightman A et al. *JAMA Network Open*.

1039 - Revision rotator cuff repair
1042 - Rotator cuff repair with tendon graft
1043 - Circumferential glenohumeral stabilization
1045 - Major release glenohumeral joint (for arthrofibrosis/adhesive capsulitis)
1050 - Joints, arthroscopy (with or without biopsy), large joint
1074 - Joints, atherectomy, excision of lumbar intervertebral disc
1080 - Arthroscopy knee joint
1100 - Morsellized bone graft (allograft, not synthetic bone graft, to one or more sites)
1103 - Joints, synovectomy, ankle
1104 - Joints, synovectomy, wrist
1105 - Orthopedics, cervical, 2 - C7 two vertebrae
1111/1114/1115 - Orthopedics, decompression (cervical, thoracic, lumbar)
1121/1122/1123 - Orthopedics, posterior or posterolateral fusion (cervical, thoracic, lumbar)
1126 - Orthopedics, intervertebral disc replacement
1133 - Orthopedics, bone graft, onlay graft for posterior lateral fusion
1139 - Orthopedics, odontoid fracture, open vertebroplasty
1144 - Arthroplasty, finger
1168/1170/1177 - Joints, arthrodesis, fusion of joint, with or without bone graft
1171 - Artificial disc insertion
1180 - Radial head arthroplasty only with implant
1182 - Primary total arthroplasty - 2 or 3 components
1184 - Joints, atherectomy, other joints, lower extremity
1185 - Joints, arthrodesis, foot, triple arthrodesis, unilateral
1191 - Joints, atherectomy, acromionectomy
1200 - Shoulder, total arthroplasty with glenoid and humeral components
1201/1202 - Suture or repair of joint capsule (capsulorrhaphy), shoulder
1209 - Removal of hardware (plates, pins, wires, screws, etc.) from the spine
1210 - Complete spinal duraplasty requiring application of a graft for tears
1211-1218/1220 – Suture or repair of joint capsule (capsulorrhaphy), knee
1252 - Midfoot joint arthrodesis
1292/1297/1301/1357/1363/1378 - Dislocation, open reduction
1390 - Arthroscopy, elbow with therapeutic intervention
1402 - Total knee arthroplasty, with patellar resurfacing
1408/1409 - Revision knee arthroplasty with removal and replacement
1415 - Total hip arthroplasty
1431 - Bursae, excision of bursa, olecranon
1452 - Muscles, gastrocnemius, recession, at calf
1471 - Arthroscopy, hip with therapeutic intervention
1511/1519 - Incision, tendon sheath, drainage for acute tenosynovitis
1525 - Incision, humerus, lateral epicondyle, stripping for "tennis elbow"
1531 - Incision, iliotibial band, division, open operation
1535 - Incision, tenotomy, corrective, single digit, subcutaneous
1552 - Excision, tendon or fibrous sheath, excision of lesion, digits only
1553 - Excision, tendon or fibrous sheath, excision of lesion, other locations
1570/1573/1574 - Excision, fasciotomy
1580/1582/1583 - Extensor tendon, repair or suture
1585/1586 - Transfer or transplant, or free graft
1589 - Tendon, lengthening or shortening
1593 - Transfer or transplant, or free graft, multiple transfer, for peripheral nerve palsy
1612/1613 - Fascial graft, free, for reconstruction of tendon or repair bowstring
1632/1633/1635/1640/1641/1654/1659/1661 - Repair, patellar advancement
1670 - Peritalar arthroscopy, regardless of portals used

© 2025 Wightman A et al. *JAMA Network Open*.

1671 - Second peritalar joint arthroscopy
1725/1740/1742/1743/1767/1802/1819 - Amputation
1820 - Arthroscopic radiocarpal joint
1893 - Plaster casts, cylinder cast (ankle to thigh)
1897 - Plaster casts, ambulatory leg cast, long leg
2593 - Wound or injury of major artery, repair peripheral vessels, suture
3577 - Laparotomy for trauma
3631/3633/3635 - Hernias, inguinal
3660/3661 - Hernias, ventral
3663 - Hernias, epigastric
3666 - Hernias, umbilical
5200 - Laminectomy for decompression of spinal cord, bilateral, first level
5203 - Laminotomy, intervertebral discs, excision anterior approach, cervical
5205 - Laminectomy, for decompression of the spinal cord, unilateral, first level
5284/5286/5287/5292/5293 - Suture of nerves
5353 - Intraplexal intrafasicular nerve transfers
5356 - Extraplexal intrafasicular peripheral nerve transfers

© 2025 Wightman A et al. *JAMA Network Open*.
