# Supplementary material for: Workplace Injury and Mental Health Outcomes
Source: JAMA Netw Open. 2025 Feb 13;8(2):e2459678. doi: 10.1001/jamanetworkopen.2024.59678 (PMC11826355; doi:10.1001/jamanetworkopen.2024.59678)
Supplement: Supplement 2. — Data Sharing Statement [file jamanetwopen-e2459678-s002.pdf]

Data Sharing Statement

Wightman. Workplace Injury and Mental Health Outcomes. *JAMA Netw Open*. Published February 13, 2025. doi:10.1001/jamanetworkopen.2024.59678

Data
Data available: No

Additional Information
Explanation for why data not available: Data cannot be shared publicly due to privacy and access restrictions through the Manitoba Centre for Health Policy (https://umanitoba.ca/manitoba-centre-for-health-policy/data-repository) and the Provincial Health Research Privacy Committee (https://www.rithim.ca/phrpc-submission-requirements).
